# Mediating Effect of Humanism on the Relationship between Task Performance Competence and Holistic Nursing Competence for Clinical Nurses

**DOI:** 10.3390/healthcare11131953

**Published:** 2023-07-06

**Authors:** Junghee Yu, Taehui Kim, Hyesun Kim

**Affiliations:** 1 Department of Nursing, Chung Cheong University, Chungbuk 28171, Republic of Korea; jhyu@ok.ac.kr; 2 Department of Nursing, Joongbu University, Chungnam 32713, Republic of Korea

**Keywords:** clinical competence, holistic nursing, humanism, task performance and analysis, nurses

## Abstract

This is a cross-sectional descriptive study that investigates the mediating effect of humanism on the relationship between task performance and holistic nursing competence among clinical nurses. The participants were nurses with more than one year of work experience in general hospitals in South Korea, recruited using convenience sampling. A total of 227 data samples were collected. A self-reported questionnaire including the task performance competence scale, holistic nursing competence scale, and humanism scale was used for the survey. Data were analyzed using the *t*-test, analysis of variance, Pearson’s correlation coefficients, and hierarchical multiple regression after checking for normal distribution. The results showed that task performance competence, holistic nursing competence, and humanism differed according to characteristics such as gender, age, educational level, marital status, position, length of career, and job and salary satisfaction. Task performance competence was positively correlated with holistic nursing competence and humanism. A positive correlation was also observed between holistic nursing competence and humanism. A partial mediating effect of humanism in the relationship between task performance competence and holistic nursing competence was confirmed. Thus, to increase nurses’ holistic nursing competence, it is necessary to improve task performance competence and formulate a continuous and repetitive education program that includes humanism.

## 1. Introduction

### 1.1. Background

Nursing behavior comprises activities aimed at promoting the healing and well-being of care recipients, and the fundamental principles of nursing remain unchanged over time [1]. However, nursing roles and functions have gradually evolved to reflect changes in the social environment. As modern society continues to rapidly age, there has been a significant rise in chronic diseases and the complexity of illnesses [2]. Consequently, there is growing interest in the quality of medical care provided by hospitals. Nurses constitute the largest proportion of hospital personnel and are key figures that impact patient outcomes (e.g., mortality, complications, and infections) and determine the quality of the medical services delivered [3]. In 2013, an integrated nursing service was introduced into the Korean national medical system to reduce the workload of caregivers and to provide high-quality medical services [4]. With the expansion of integrated nursing services, particularly at general hospitals where patients tend to have more severe conditions, the demand for patient-centered and high-quality nursing care is increasing.

The Fourth Industrial Revolution resulted in robots and AI replacing various aspects of life, including nursing care, such as measuring vital signs or blood sugar levels [4]. Thus, holistic care—which encompasses qualitative aspects such as effective communication, understanding, and empathy—has become more vital than direct care delivered by nurses [5]. Holistic nursing is a multidimensional approach that prioritizes health promotion and encompasses nursing care that addresses patients’ physical, mental, social, and spiritual needs [6]. Holistic nursing competence refers to nurses’ ability to deliver effective nursing care with a professional attitude, values, knowledge, skills, and responsibility, while taking a holistic view of patients [7]. To achieve holistic nursing competence, the ability to execute the tasks fundamentally necessary for nursing is essential.

To deliver high-quality nursing care, it is imperative to fulfill patients’ needs and possess the requisite professional knowledge and nursing skills to effectively solve problems. The performance of nursing tasks encompasses the various activities undertaken by nurses to provide patient care [2]. However, the assessment of nursing task performance is crucial in quality management because, in addition to basic skills, ethical conduct and performance are evaluated, including nurses’ attitudes when delivering accurate nursing care [3]. Nursing performance is a common indicator used to evaluate task performance and determine how well nurses execute tasks in hospitals [2]. Although evidence-based nursing practices form the foundation of holistic nursing, they are inadequate to deliver true holistic care. Holistic nursing entails identifying the problems that arise when patients’ physical, socioeconomic, environmental, educational, emotional, and psychological needs are not met [8]. Nurses require a humanistic approach to resolve diverse problems faced by patients.

Humanism entails recognizing human dignity as being of the utmost value and striving for the well-being and welfare of humanity, irrespective of differences in race, nationality, or religion [9]. In nursing practice, nurses’ humanistic approaches are integrated into the value of altruism and are deemed crucial for holistic care provision [10]. Holistic nursing commences with nurses’ self-awareness and advances to empathy for patients, encompassing intuition and subjectivity [6]. Holistic nursing entails understanding patients’ circumstances and experiences from their perspective and offering nursing care based on expertise, creativity, and empathy [6]. Hence, humanism provides a basis for nurses’ compassion and understanding of patients as they offer nursing care based on their expertise. A study demonstrated that humanism affects the improvement of nursing service quality [11].

To provide high-quality, patient-centered nursing care, it is essential to enhance nurses’ competence in providing holistic care [12]. Self-compassion, an essential element of humanism, is closely related to nurses’ task performance competence, and high task performance can help improve their self-awareness [13]. High task performance can increase job satisfaction [14] and commitment [15]. Effective communication is critical for nurses to be fully immersed in their jobs and can also impact their relationships with patients or guardians. When nurses perform well at their jobs, their humanism may be positively influenced, which could enhance their ability to empathize with patients. Hence, nurses’ ability to effectively perform their jobs can impact their humanistic values, which, in turn, can enhance their holistic nursing competence. In other words, if humanism is improved due to nurses’ task performance competence, the holistic nursing competency can also be improved due to the improvement of humanism. There is a need to explore whether this phenomenon actually occurs in nurses; however, foreign studies on humanism and nursing have mainly adopted qualitative approaches [6,10]. Korean studies have focused on exploring the correlations between emotional intelligence, communication skills, and end-of-life nursing performance with holistic nursing or humanism [16,17]. Despite the significance of holistic nursing care in improving nurses’ task performance, research on the impact of task performance competence and humanism on nurses’ holistic nursing care is lacking. This study aimed to investigate the role of humanism in the relationship between task performance competence and holistic nursing competence, thereby providing valuable insights into developing programs that enhance clinical nurses’ holistic nursing competence.

### 1.2. Study Purpose

This study aimed to evaluate the mediating effect of humanism on the relationship between task performance competence and holistic nursing competence of clinical nurses.

## 2. Materials and Methods

### 2.1. Study Design

This study explored the mediating effect of humanism on the relationship between clinical nurses’ task performance competence and holistic nursing competence.

### 2.2. Participants

This study targeted nurses from three general hospitals located in D and S cities in South Korea. The specific selection criteria were as follows: (1) nurses currently and primarily working in patient care, (2) nurses with more than one year of clinical experience, and (3) nurses who understood the purpose of the study and voluntarily decided to participate. Clinical experience of more than one year was required because it takes more than one year for nurses to adapt to work in the clinical field [11]. Data were collected from 230 nurses in consideration of the recovery rate and response fidelity, considering that a sample size of 200 is appropriate when the number of observed variables is less than 12 [18]. The data of 227 participants were used for the analysis, excluding those of 3 participants with insufficient responses.

### 2.3. Measures

A total of 114 items, including general characteristics (10 items), the task performance competence scale (35 items), the holistic nursing competence scale (36 items), and the humanism scale (15 items), were surveyed.

#### 2.3.1. Task Performance Competence Scale

Task performance competence was measured using the Performance Rating Scale developed by Paik et al. [1]. This tool consists of 4 areas (knowledge, attitude, performance, and ethics) and 35 questions. It is measured on a 5-point Likert scale, and the higher the total score, the higher the task performance competence. Cronbach’s α at the time of tool development was 0.91 [1], and that in this study was 0.97.

#### 2.3.2. Holistic Nursing Competence

Holistic nursing competence was measured using the Holistic Nursing Competence Scale developed by Takase and Teraoka [7] and translated into Korean by Seo et al. [12]. This tool consists of 36 items measured on a 7-point Likert scale. The higher the score, the higher the nurses’ holistic nursing competence. Cronbach’s α of the tool was 0.96 in Seo et al.’s [12] study, and that in this study was 0.96.

#### 2.3.3. Humanism

Humanism was measured using the Humanism Short Scale developed by Nilsson [19] and translated into Korean by Lee and Seo [20]. This tool consists of 15 items measured on a 7-point Likert scale. The total score for each item was used, and the higher the total score, the higher the degree of humanism. Cronbach’s α at the time of tool development was 0.93 [20], and that in this study was 0.96.

### 2.4. Data Collection

This study was approved by the Institutional Review Board of Joongbu University (JIRB-2022112201-01-221226) and was conducted between 1 January and 28 February 2023. The self-report questionnaire used in this study included questions related to general and clinical characteristics, task performance competence, holistic nursing competence, and humanism. After explaining the purpose and contents of the study to the nursing departments of each hospital, the questionnaire was distributed to nurses through the head nurses of each ward. The participants were informed about the survey’s background and purpose, as well as the anonymity and confidentiality of their responses. They were also informed that the results would be used solely for research purposes and destroyed at the end of the study. The participants were given a small gift as an incentive to participate. The completed questionnaires were collected separately from the consent form, stored in a locked cabinet, and anonymously computer-coded for analysis.

### 2.5. Statistical Analysis

The data collected for this study were analyzed using IBM SSPS 24.0 (IBM Corp., Armonk, NY, USA). The specific analysis methods were as follows.

First, the general characteristics and degree of task performance competence, holistic nursing competence, and humanism were analyzed using descriptive statistics. The normality of the analysis data was calculated by skewness and kurtosis.

Second, the differences in task performance competence, holistic nursing competence, and humanism according to general characteristics were analyzed using an independent *t*-test and one-way analysis of variance, and the Scheffé test was used for post hoc analysis.

Third, the correlations between task performance competence, holistic nursing competence, and humanism were analyzed using Pearson’s correlation coefficients.

Fourth, the mediating effect of humanism on the relationship between task performance competence and holistic nursing competence was analyzed using hierarchical multiple regression. In the first step of the regression analysis, it was confirmed whether the independent variable regressed to the mediating variable, and in the second step, it was confirmed whether the independent variable regressed to the dependent variable. In the final step, it was confirmed whether the independent variable and mediating variable regressed to the dependent variable. At this time, the effect of the independent variable should be smaller in the third stage than in the second stage. The significance of the mediating effect of humanism on the relationship between task performance competence and holistic nursing competence was analyzed using the Sobel test. Reliability was measured using Cronbach’s α.

## 3. Results

### 3.1. General Characteristics

Most participants (92.1%) in this study were female, with an average age of 30.1 (±4.82) years, and the largest age group (56.8%) was under 29 years. Most of the nurses (85.5%) had a Bachelor’s degree (85.5%), 9.3% reported religious activities, and 24.2% were married. Most participants worked in general wards (61.2%) and were staff nurses (97.4%). The average nursing career was 6.04 (±5.08) years, with 4 to 10 years being the most common (43.6%). Job satisfaction was the highest at 59.5%, whereas dissatisfaction with wages was the highest at 66.5%. The average task performance competence score for the participants in this study was 3.74 (±0.46) points, and the holistic nursing competence score was 4.91 (±0.84) points. The average humanism score was 4.36 (±1.02) points (Table 1). When examining skewness and kurtosis to determine whether the variables were normal, skewness was −0.59~0.60, and kurtosis was confirmed to be −0.72~0.62. It was confirmed that normality was secured with the skewness of all variables below the absolute value of 3 and the kurtosis below the absolute value of 10.

### 3.2. Differences in Task Performance Competence, Holistic Nursing Competence, and Humanism According to General Characteristics

In this study, task performance competence differed according to the participants’ general characteristics, such as sex (*t* = −3.33, *p* = 0.003), age (*F* = 5.14, *p* = 0.007), education level (*F* = 7.50, *p* < 0.001), marital status (*t* = −2.81, *p* = 0.006), position (*t* = −2.66, *p* = 0.033), length of career (*F* = 7.22, *p* < 0.001), job satisfaction (*F* = 4.63, *p* = 0.011), and salary satisfaction (*F* = 3.89, *p* = 0.022). Female participants performed better than male participants, and performance was higher among those aged 40 years or older than among those aged 29 years or younger. Participants with a Master’s degree performed better than those with a Bachelor’s degree, and married participants performed better than unmarried participants. The nurses in charge showed higher task performance competence than general nurses. Those with a career of more than 11 years showed higher task performance competence than those with a shorter career. Additionally, those who were satisfied with their jobs and salaries showed higher task performance competence than those who were dissatisfied or had average satisfaction.

The study found significant differences in holistic nursing competence based on the participants’ general characteristics. Gender (*t* = −2.28, *p* = 0.032), age (*F* = 10.94, *p* < 0.001), educational level (*F* = 6.61, *p* = 0.002), marital status (*t* = −3.89, *p* < 0.001), position (*t* = −2.77, *p* = 0.006), and length of career (*F* = 7.37, *p* < 0.001) contributed to the differences in holistic nursing competence. Specifically, female participants scored higher than male participants, and those aged 30 years or older scored higher than those aged 29 years or younger. Those with a Master’s degree scored higher than those with a three-year nursing school education or Bachelor’s degree, and married nurses scored higher than unmarried nurses. Additionally, nurses in charge scored higher than general nurses, and those with more than 11 years of experience scored higher than those with less than 10 years of experience.

Gender (*t* = −3.32, *p* = 0.001), age (*F* = 27.85, *p* < 0.001), educational level (*F* = 9.67, *p* < 0.001), marital status (*t* = −4.21, *p* < 0.001), and length of career (*F* = 14.85, *p* < 0.001) were associated with the differences in humanism among the participants. Female participants exhibited higher levels of humanism than men, and the level of humanism increased with age. Those with a three-year nursing school education and Master’s degree showed higher levels of humanism than those with only a Bachelor’s degree. Additionally, humanism was higher among married than unmarried individuals. Regarding the length of career, those with 4 to 10 years of experience or less showed higher levels of humanism than those with less than 3 years of experience, and those with 11 or more years of experience showed higher levels of humanism than those with 4 to 10 years of experience or less (see Table 2).

### 3.3. Correlations between Task Performance Competence, Holistic Nursing Competence, and Humanism

Task performance competence was positively correlated with holistic nursing competence (*r* = 0.71, *p* < 0.001) and humanism (*r* = 0.61, *p* < 0.001). A positive correlation was also observed between holistic nursing competence and humanism (*r* = 0.47, *p* < 0.001) (Table 3).

### 3.4. Mediating Effect of Humanism on Relationship between Task Performance Competence and Holistic Nursing Competence

A three-stage regression analysis was conducted to investigate the mediating effect of humanism on the relationship between task performance competence and holistic nursing competence. Before examining the mediating effect, the independence of the residuals was checked by verifying the Durbin–Watson value, which ranged from 1.58 to 1.86, indicating no autocorrelation of the dependent variable. Multicollinearity was also checked, and all variables had tolerance limits below 1.0 and variance expansion coefficients between 1.00 and 1.28, indicating no issues with multicollinearity. Thus, the regression model was considered suitable for analysis.

**Figure 1:**
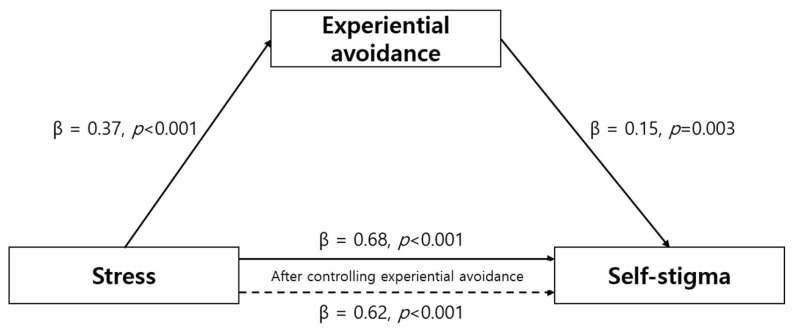
Mediating effect of humanism on the relationship between task performance and holistic nursing competence.

The regression analysis showed that task performance competence significantly affected humanism (β = 0.47, *p* < 0.001) in Step 1. In Step 2, task performance competence significantly affected holistic nursing competence (β = 0.71, *p* < 0.001). In Step 3, both task performance competence and humanism affected holistic nursing competence (β = 0.54, *p* < 0.001; β = 0.36, *p* < 0.001), indicating a partial mediating effect (*F* = 174.09, *p* < 0.001). This implies that task performance competence has both direct and indirect effects on holistic nursing competence through its impact on humanism. The explanatory power of the regression analysis was 60.5%. The Sobel test confirmed the significance of the mediating effect of humanism (*Z* = 3.59, *p* < 0.0014) on the relationship between task performance competence and holistic nursing competence (Table 4, Figure 1).

## 4. Discussion

This study investigated the mediating effect of humanism on the relationship between task performance competence and holistic nursing competence among clinical nurses in South Korea. With the increasing significance of patient-centered nursing, which emphasizes understanding and empathy for patients, it is necessary to explore the role of humanism in facilitating this approach.

Clinical nurses’ task performance competence was evaluated at 3.74 points (range: 1–5 points), with differences based on gender, age, education level, marital status, position, length of career, and job satisfaction. In contrast, Kim and Han [13] reported higher task performance competence among clinical nurses at 4.07 points, with differences based on age, marital status, religion, position, and length of career, but not education level. The disparity in task performance competence scores could be attributed to differences in clinical settings and participant characteristics. Specifically, this study focused on ward and intensive care unit nurses requiring continuous patient management skills, whereas Kim and Han’s study [13] included emergency and operating rooms as well as head nurses. Kim and Han [13] reported that emergency, operating, and head nurses demonstrated high task performance competence scores. In contrast, Kim and Lee [13] reported a task performance competence score of 2.74 points (range: 1–5 points) among university hospital nurses, which is lower than that reported in this study. This difference is because Kim and Lee [14] included nurses with less than one year of total career experience, and the participants were recruited from a university hospital, unlike this study or that of Kim and Han [13]. As age and length of career are related to variables such as education, position, and marital status, differences in task performance competence can be considered within the same context [21]. Therefore, to improve the quality of nursing services through increased task performance competence in clinical nurses, it is necessary to provide institutional support that enables uninterrupted work for nurses and systematic educational programs targeting those with less career experience.

In this study, clinical nurses had an average holistic nursing competence score of 4.91 points (range: 1–7 points), with differences according to gender, age, education level, marital status, position, and length of career. This average score was similar to the 4.94 points reported in a validity study of the holistic nursing competence scale in Korea, but higher than the average score of 4.22 points reported by Takase and Teraoka [7], who developed the tool. The differences observed in holistic nursing competence were similar to those observed in task performance competence and can be attributed to the belief that holistic care begins with self-understanding and self-reflection [6,22]. As nurses gain more experience and reflect on their work, their understanding of humans and their beliefs about caring may increase. However, since this study relied on nurses’ self-reported questionnaires to measure holistic nursing, there may be differences from what patients perceive. Therefore, additional research is needed to identify the factors that affect nurses’ holistic care and their effects on various aspects.

The participants of this study had an average humanism score of 4.36 points (range: 1–7 points), and there were differences in the scores according to gender, age, educational level, marital status, and length of career. In a previous study of nursing students in Korea [23], the humanism score was higher at 5.49 points, and there was no difference based on gender, age, or education level. However, differences were observed based on the service experiences of older adults. Humanism arises from interpersonal relationships [24] and can be enhanced when nurses recognize and respect patients as individuals. There is a lack of studies examining the factors that affect nurses’ humanism. Additionally, considering the humanism, nurses need to think about how to improve it. Therefore, various approaches and multidimensional studies on humanism are needed in the future.

In this study, a positive correlation was observed between clinical nurses’ task performance competence, holistic nursing competence, and humanism. Humanism was found to play a partial mediating role in the relationship between task performance competence and holistic nursing competence. Task performance competence is defined as the ability to safely provide nursing care while following ethical principles and integrating knowledge, skills, abilities, and judgment under various conditions [25]. Thus, improving nurses’ task performance competence can increase their humanism as they recognize the patient as an individual while providing nursing care based on ethical principles. A humanistic approach to nursing can help them understand the personal values of their profession, enhance their professional skills, and improve their job satisfaction [24]. Holistic nursing is characterized by a “mind–body–mind–emotions–environment” approach that seeks to understand the patient’s condition and experience from their perspective, and provide nursing care through creativity, empathy, and expertise [6]. Humanism helps nurses understand patients better and develop empathy and altruism toward them, which enables them to understand patients better and provide a more solid foundation of care. Therefore, when designing educational programs to enhance the holistic nursing competence of clinical nurses, it is crucial to prioritize improving task performance competence and include humanism as a key element.

This study has some limitations. First, convenience sampling was conducted with only a subset of clinical nurses in one district. Therefore, caution must be exercised before the findings of this study are generalized to other populations. Further studies targeting a larger number of nurses should be conducted to obtain more robust results. In addition, since a self-reporting questionnaire was used, and there is an endogeneity problem, in the future, direct observational research needs to be conducted to supplement this. Nevertheless, this study is significant because it shed light on the relationship between task performance competence, holistic nursing competence, and humanism among clinical nurses. Humanism partially mediated the effect of task performance competence on holistic nursing competence. These findings can serve as a foundation for future research aimed at improving clinical nurses’ holistic nursing competence.

## 5. Conclusions

This study aimed to establish foundational data for enhancing clinical nurses’ holistic nursing competence by examining the mediating effect of humanism on the relationship between task performance competence and holistic nursing competence. Significant positive correlations were observed between clinical nurses’ task performance competence, holistic nursing competence, and humanism. Additionally, humanism was found to partially mediate the relationship between task performance competence and holistic nursing competence. These findings imply that improving task performance competence is a prerequisite for enhancing clinical nurses’ holistic nursing competence and that further improvements in humanism can also contribute to this goal. Based on these findings, humanism should be included in educational programs aimed at improving clinical nurses’ holistic nursing competence. Improving task performance competence and incorporating elements of humanism will enhance clinical nurses’ holistic nursing competence.

## Figures and Tables

**Table 1:** Participants’ general characteristics (N = 227).

Characteristics	Categories	M ± SD or N (%)	Min–Max
Sex	Male	18 (7.9)	
	Female	209 (92.1)	
Age (years)		30.1 ± 4.82	23.0–53.0
	≤29	129 (56.8)	
	30–39	84 (37.0)	
	≥40	14 (6.2)	
Educational level	3-year nursing school	23 (10.1)	
	Bachelor’s degree	194 (85.5)	
	Master’s degree or higher	10 (4.4)	
Having a religion	Yes	21 (9.3)	
	No	206 (90.7)	
Marital status	Yes	55 (24.2)	
	No	172 (75.8)	
Department	General ward	139 (61.2)	
	Intensive care unit	88 (38.8)	
Position	Staff nurse	221 (97.4)	
	Charge nurse	6 (2.6)	
Length of career (years)		6.04 ± 5.08	1–31.0
	>2	37 (16.3)	
	2–3	53 (23.3)	
	4–10	99 (43.6)	
	≥11	38 (16.7)	
Job satisfaction	Dissatisfaction	39 (17.2)	
	Moderate	135 (59.5)	
	Satisfaction	53 (23.3)	
Salary satisfaction	Dissatisfaction	151 (66.5)	
	Moderate	65 (28.6)	
	Satisfaction	11 (4.8)	
Task performance competence		3.74 ± 0.46	2.37–5.00
Holistic nursing competence		4.91 ± 0.84	2.78–7.00
Humanism		4.36 ± 1.02	2.73–7.00

M = Mean; SD = Standard deviation; Min = Minimum; Max = Maximum.

**Table 2:** Differences in task performance competence, holistic nursing competence, and humanism according to general characteristics (N = 227).

Characteristics	Categories	Task Performance Competence			Holistic Nursing Competence			Humanism		
		M ± SD	*t* or *F*	*p* Scheffe	M ± SD	*t* or *F*	*p* Scheffe	M ± SD	*t* or *F*	*p* Scheffe
Sex	Male	3.42 ± 0.43	−3.33	0.003	4.54 ± 0.70	−2.28	0.032	3.61 ± 0.68	−3.32	0.001
	Female	3.77 ± 0.46			4.94 ± 0.84			4.43 ± 1.01		
Age (years)	≤29 ^a^	3.67 ± 0.42	5.14	0.007 a < c	4.70 ± 0.79	10.94	<0.001 a < b,c	4.01 ± 0.76	27.85	<0.001 a < b < c
	30–39 ^b^	3.81 ± 0.50			5.15 ± 0.85			4.70 ± 1.12		
	≥40 ^c^	4.03 ± 0.50			5.42 ± 0.62			5.58 ± 1.02		
Educational level	3-year nursing school ^a^	3.85 ± 0.41	7.50	0.001 b < c	5.00 ± 0.81	6.61	0.002 a,b < c	4.81 ± 1.09	9.67	<0.001 b < a,c
	Bachelor’ degree ^b^	3.71 ± 0.46			4.85 ± 0.83			4.25 ± 0.97		
	≥Master’s degree or higher ^c^	4.25 ± 0.43			5.81 ± 0.47			5.44 ± 0.82		
Having a religion	Yes	3.81 ± 0.27	0.67	0.499	4.94 ± 0.79	0.19	0.846	4.67 ± 0.93	1.60	0.121
	No	3.74 ± 0.48			4.91 ± 0.84			4.33 ± 1.02		
Marital status	Yes	3.91 ± 0.53	−2.81	0.006	5.29 ± 0.82	−3.89	<0.001	4.85 ± 1.16	−4.21	<0.001
	No	3.69 ± 0.43			4.79 ± 0.81			4.21 ± 0.92		
Department	General ward	3.73 ± 0.44	−0.30	0.761	4.87 ± 0.88	0.88	0.375	4.35 ± 1.02	0.22	0.822
	Intensive care unit	3.73 ± 0.48			4.97 ± 0.77			4.38 ± 1.01		
Position	Staff nurse	3.74 ± 0.47	−2.66	0.033	4.89 ± 0.83	−2.77	0.006	4.34 ± 1.01	−2.03	0.094
	Charge nurse	3.96 ± 0.18			5.84 ± 0.22			5.15 ± 0.96		
Length of career (years)	≤1 ^a^	3.59 ± 0.45	7.22	<0.001 a,b,c < d	4.56 ± 0.75	7.37	<0.001 a,b,c < d	3.76 ± 0.69	14.85	<0.001 a,b < c < d
	2–3 ^b^	3.71 ± 0.41			4.77 ± 0.76			4.08 ± 0.70		
	4–10 ^c^	3.71 ± 0.44			4.94 ± 0.83			4.45 ± 1.04		
	≥11 ^d^	4.04 ± 0.50			5.39 ± 0.83			5.10 ± 1.09		
Job satisfaction	Dissatisfaction ^a^	3.66 ± 0.49	4.63	0.011 a,b < c	4.95 ± 0.96	2.40	0.93	4.51 ± 1.09	1.20	0.301
	Moderate ^b^	3.70 ± 0.45			4.82 ± 0.81			4.39 ± 1.06		
	Satisfaction ^c^	3.91 ± 0.44			5.12 ± 0.78			4.19 ± 0.82		
Salary satisfaction	Dissatisfaction ^a^	3.74 ± 0.44	3.89	0.022 a,b < c	4.90 ± 0.84	2.38	0.094	4.30 ± 0.98	1.01	0.364
	Moderate ^b^	3.70 ± 0.50			4.86 ± 0.84			4.44 ± 1.08		
	Satisfaction ^c^	4.12 ± 0.35			5.44 ± 0.63			4.69 ± 1.15		

M = Mean; SD = Standard deviation. Superscripts indicate each category. To denote result of post test (scheffe test), a letter was added in superscript after each category.

**Table 3:** Correlations between task performance competence, holistic nursing competence, and humanism (N = 227).

	1 r (*p*)	2 r (*p*)	3 r (*p*)
1. Task performance competence	1		
2. Holistic nursing competence	0.71 (<0.001)	1	
3. Humanism	0.61 (<0.001)	0.47 (<0.001)	1

**Table 4:** Mediating effect of humanism on the relationship between task performance competence and holistic nursing competence (N = 227).

Step	Independent Variables	Dependent Variables	B	SE	β	*t* (*p*)	Adj. R^2^	*F* (*p*)
1	TPC	Humanism	1.03	0.12	0.47	8.03 (<0.001)	0.219	64.52 (<0.001)
2	TPC	HNC	1.28	0.08	0.71	15.27 (<0.001)	0.507	233.18 (<0.001)
3	TPC	HNC	0.98	0.08	0.54	11.48 (<0.001)	0.605	174.09 (<0.001)
	Humanism	HNC	0.29	0.04	0.36	7.54 (<0.001)		
			Sobel test; *Z* = 3.59 (*p* < 0.001)					

Adj. = Adjusted; SE = Standard error; TPC = Task performance competence; HNC = Holistic nursing competence.

## Data Availability

Not applicable.
